# Is the Physiological Composition of the Vaginal Microbiome Altered in High-Risk HPV Infection of the Uterine Cervix?

**DOI:** 10.3390/v14102130

**Published:** 2022-09-27

**Authors:** Tomas Rokos, Veronika Holubekova, Zuzana Kolkova, Andrea Hornakova, Terezia Pribulova, Erik Kozubik, Kamil Biringer, Erik Kudela

**Affiliations:** 1 Department of Gynecology and Obstetrics, Jessenius Faculty of Medicine, Comenius University in Bratislava, Kollarova 2, 03601 Martin, Slovakia; 2 Department of Molecular Oncology and Diagnostics, Biomedical Center Martin, Jessenius Faculty of Medicine, Comenius University in Bratislava, Mala Hora 4C, 03601 Martin, Slovakia

**Keywords:** human viruses, HPV, vaginal microbiome, cervical precancerosis

## Abstract

Background: Cervical cancer is the fourth most common malignancy and fourth leading cause of cancer death in women worldwide. More than 99.7% of cases are caused by human papillomavirus (HPV), while HPV types 16 and 18 cause over 70% of all cervical cancer cases. In this preliminary study, we aimed to investigate the presence of HPV infection and diversity of bacteria associated with bacterial vaginosis. Methods: Cervical swabs (*n* = 21) taken from women aged 21–47 years, in seventeen cases, with different degrees of cervical abnormality, and from four healthy women, were tested for the presence of HPV DNA, as well as the bacterial strains associated with bacterial vaginosis, using the real-time PCR method. Results: HPV16 was the dominant genotype in 53% (9/17) of patients with confirmed precancerous lesions (ASCUS, LSIL, and HSIL). In specimens with confirmed cytological abnormalities and hrHPV infection, we detected a wide diversity of microbes, while the most common species were *Gardnerella vaginalis*, *Atopobium vaginae*, *Prevotella bivia*, *Ureaplasma parvum*, *Ureaplasma urealyticum*, *Leptotrichia amnionii*, *Bacteroides ureolyticus*, and *Sneathia sanguinegens*. The presence of pathogens did not differ, depending on the degree of precancerous lesions or HPV type. Conclusion: In our work, HPV16 dominated in patients with cervical precancerous lesions. We also suggest an increased bacterial diversity of the vaginal microbiome in patients with cervical lesions, for which the HPV virus is largely responsible.

## 1. Introduction

Cervical cancer is the fourth most common malignancy and fourth leading cause of cancer death in women worldwide [1]. More than 99.7% cases are caused by human papillomavirus (HPV), while HPV infection is the most common viral infection of the reproductive tract [2]. The International Agency for Research on Cancer classifies HPV types according to their carcinogenic potential into four groups, with 12 representing high-risk HPV types (hrHPV) [3]. HPV oncoproteins E6 and E7 cause dysfunction of tumor suppressor proteins and dysregulation of the cell cycle. In hrHPV infection, these changes can lead to neoplastic transformation of the affected tissue. Dysregulations of gene expressions, such as increased phosphoglycerate dehydrogenase gene expression or overexpression of some subsets of stemness pathway genes, may also be involved in this transformation [4,5]. Untreated lesions can eventually develop into invasive disease [6]. Worldwide, HPV types 16 and 18 cause over 70% of all cervical cancer cases, while the highest prevalence is observed in countries in Africa and Oceania. In general, the highest incidence of HPV infection is in the population of younger women, with the peak incidence occurring at an age of less than 25 years and corresponding incidence decrease with an increase in age. Such a decrease is not observed in developing countries. The most frequently detected oncogenic type is HPV16, followed by HPV18, HPV31, HPV52, and HPV58 [7]. HPV is detected in 52.5% of atypical squamous cell lesions of unknown significance (ASCUS), 74.8% of LSIL lesions, and 88.9% of HSIL lesions [8]. 

The HPV virus affects up to 80% of women during their lifetimes [9]. Despite this prevalence, the development of cervical cancer is rare. In almost 90% of infected women, the virus is removed without clinical symptoms. Among the HPV types classified as high-risk by the IARC, HPV16 dominates in the Czech Republic, followed by HPV18, in the case of invasive disease. However, for diagnosed precancerous lesions, in the case of LSIL, the second most common type is HPV33; in the group of female patients with HSIL, HPV types 16 and 33 are followed by type 31 [10]. 

The human vaginal microbiome, in the right composition, contributes to the prevention of urological tract infections [11], urogenital diseases, and sexually transmitted diseases [12]. The lactic-acid-producing genus Lactobacillus plays an important role in this microbiome. Ravel J. et al. [13] examined the vaginal microbiome and vaginal pH of 396 asymptomatic, sexually active women and divided the vaginal microbiome into five groups, called community state types (CSTs). The dominant microbe of group I was *Lactobacillus crispatus*; in group II, *Lactobacillus gasseri* dominated; in group III, *Lactobacillus iners* was dominant; and *Lactobacillus jensenii* was dominant in group V. Vaginal swab examinations in the remaining 27% patients showed the dominance of strict anaerobes, including *Gardnerella*, *Prevotella*, *Megasphaera*, and *Sneathia*. The lowest pH was found in group I, while the highest pH was observed in group IV, corresponding to a higher risk of BV development [13]. 

The vaginal microbiome associated with bacterial vaginosis is a predisposing factor for sexually transmitted infections [14,15] and the development of pelvic inflammatory disease [16]. Any imbalance in the physiological settlement of the vagina increases the risk of bacterial and viral infection. Women with BV have a higher risk of the gonococcal and chlamydial [17], herpes simplex virus 2 [14], and human immunodeficiency virus 1 [15] infections. Entry of the HPV virus into the basal cells of the uterine cervix, the associated cervical intraepithelial changes, and the progression to cervical cancer are also associated with vaginal dysbiosis. The reduced abundance of lactobacilli, which are replaced by anaerobic bacteria in women with BV, decrease the protective function of lactobacilli in the vagina, thus yielding reduced production of hydrogen peroxide [18], decreased rates of sugar metabolism, increases in pH, reduced functions of local cell-mediated immunity, and decreased production of IgG and IgA immunoglobulins [19]. The production of bactericidal and bacteriostatic proteins that inhibit the growth and multiplication of pathogens is also reduced [20]. 

The aim of our work is to investigate the possible association of vaginal dysbiosis with hrHPV infection and subsequent development of precancerous lesions of the cervix. We also sought to determine the representation of HPV genotypes in the studied cohort, according to the types of precancerous lesions.

## 2. Materials and Methods

### 2.1. Patients and Sample Collection

Sampling was performed during the expert colposcopic examination of patients with cytological abnormalities. Control samples from women negative for intraepithelial lesions of the cervix were taken during a routine gynecological examination. In both cases, all participants were fully informed that anonymity is assured, why the research is being conducted, how their data will be used, and the associated risks. All participants expressed clear consent for the collection of biological material. During the expert colposcopic examination, the samples of the vaginal microbiome were taken from the posterior vaginal fornix with, and from, the area of the cervical external orificium using cytobrush. The optical examination of the cervix consisted of native and extended colposcopy, using a solution of 3% acetic acid and iodine solution (Schiller’s test), which also served to describe the cervical lesions in the case of tissue biopsy.

Cervical swabs were immersed in RNA stabilization and storage solution (Ambion, Thermo Fisher Scientific, Austin, TX, USA), transported to Biomedical Centre in Martin at room temperature, and stored at −20 °C until further analysis.

### 2.2. DNA Extraction

Cervical swabs were unfrozen, vortexed, and rinsed with phosphate buffered saline (PBS). A total of 100 μL of cervical cell suspension was employed for DNA extraction using a MasterPure Complete DNA and RNA Isolation kit (Epicentre Biotechnologies, Madison, WI, USA), according to the manufacturer’s instructions. Extracted DNA was diluted in 35 μL of TE buffer, and the quantity was measured using a Qubit dsDNS HS assay kit (Invitrogen, Thermo Fisher Scientific, Carlsbad, CA, USA). All samples were stored at −20 °C before subsequent analysis.

### 2.3. HPV Genotyping

HPV genotyping was performed using PCR targeting the L1 [21] conserved region of HPV16 and HPV18, similar to location published by Gravitt et al. [22]. The amplification of HPV genotypes was performed in a total volume of 25 μL containing 50 ng of DNA, 200 μM of dNTPs, 2.5 mM of MgCl_2_, 50 nM of each primer, and 1 U of Hot Start*Taq* DNA polymerase (Qiagen, Hilden, Germany). PCR amplicons were checked on 1.75% agarose gel stained with gel red nucleic acid gel stain (Biotium Inc., Hayward, CA, USA) and purified by NucleoSpin Gel and PCR Clean-Up kit (Macherey-Nagel GmbH and Co. KG, Duren, Germany). Reactions for the cycle sequencing of 420 bp fragments (Applied Biosystems by ThermoFisher Scientific, Vilnius, Lithuania) were purified on gel matrix (Sigma-Aldrich, St. Louis, MO, USA). The HPV genotypes were distinguished via sequencing on a 3500 genetic analyzer (Applied Biosystems, Thermo Fisher Scientific, Foster City, CA, USA) and compared to other known sequences in an online database (BLAST; National Center for Biotechnology Information).

### 2.4. Identification of Bacteria Associated with Bacterial Vaginosis

In total, 250 ng of DNA was tested via a real-time PCR to identify of microbial strains associated with bacterial vaginosis, according to the manufacturer’s instructions (Microbial DNA qPCR Array, Bacterial Vaginosis, Format C, Qiagen, Hilden, Germany). The microbial qPCR array contained assays for 42 pathogenic bacterial species, as well as control human genes (GAPDH and HBB1) [23,24], bacterial 16S rRNA and fungal ribosomal rRNA genes [25], and positive PCR control. Each reaction contained 12.5 μL of microbial qPCR master mix, primers/probe depending on the position on the plate, 11.5 μL of microbial DNA free water, and 5 ng DNA in one microliter in a total volume of 25 microliters. The identification of microbial strains was performed on 7500 fast real-time PCR system (Applied Biosystems) with thermal cycling conditions, such as initial denaturation at 95 °C for 10 min, 40 cycles with denaturation at 95 °C for 15 s, and annealing and extension at 60 °C for 2 min. All samples were analyzed in the instrument software, as recommended by the manufacturer.

## 3. Results

### 3.1. Participants’ Characteristics

Here, we present partial results for 21 patients, from a set of 152 collected samples, as sampling is still ongoing. The presented patients were between 21 and 47 years of age, with cytological abnormalities demonstrated in 17 patients (ASCUS (5), LSIL (9), and HSIL (3)). The other four women had negative cytology smears.

### 3.2. HPV Genotyping via PCR and Sequencing

HPV genotyping was performed according to the previously published method [26], thus allowing us to genotype 13 high-risk HPV genotypes belonging to alpha-7 (HPV 18, 39, 45, 59, 68, and 70) and alpha-9 (HPV 16, 31, 33, 35, 52, and 58) species [27], as well as HPV66. 

Overall, HPV 16 was the dominant genotype identified in three patients with ASCUS cytological smear abnormalities; four patients with low-grade lesions, two patients with high-grade lesions, and one patient negative for an intraepithelial lesion or malignancy (NILM) were infected with this type. One patient with a low-grade lesion was infected simultaneously with two types, HPV18 and HPV66. Among the remaining samples, in the ASCUS lesion category, one patient was infected with HPV33, and one patient was infected with HPV66. One patient with an LSIL lesion was infected with HPV66, and another LSIL patient was infected with HPV70. The remaining patient with an HSIL lesion was infected with HPV52. Three NILM participants and two LSIL patients were HPV negative. The results are summarized in Table 1.

### 3.3. Composition of the Vaginal Microbiome

In all patients with confirmed cytological smear abnormalities, pathogens disrupting the physiological composition of the vaginal microflora were present in the vaginal swabs. These pathogens comprised a spectrum of various microorganisms, such as *Gardnerella vaginalis*, *Atopobium vaginae*, *Leptotrichia amnionii*, *Sneathia sanguinegens*, *Parvimonas micra*, *Prevotella melaninogenica*, *Prevotella disiens*, *Prevotella intermedica*, *Prevotella nigrescens*, *Prevotella bivia*, *Streptococcus mitis*, *Mycoplasma hominis*, *Aerococcus christensenii*, *Fusobacterium nucleatum*, *Corynebacterium aurimucosum*, *Streptococcus intermedius*, *Ureaplasma parvum*, *Ureaplasma urealyticum*, *Campylobacter gracilis*, *Treponema denticola*, *Bacteroides ureolyticus*, *Mobiluncus curtisii*, and *Candida albicans*.

The aforementioned pathogens were represented differently, as some bacterial species occurred in a larger number of patients. *Gardnerella vaginalis* was confirmed in 75% of HPV-positive patients, but it was confirmed in patients with every type of precancerous lesion detected in our cohort. *Gardnerella vaginalis* was also confirmed in 80% HPV-negative women. *Prevotella bivia* and *Atopobium vaginae* were detected, respectively, in 44% and in 50% of HPV-positive patients, while *Prevotella bivia* was confirmed in of 40% HPV-negative participants. *Ureaplasma parvum/urealyticum* infection was observed in 56% of HPV positive cases and 60% of HPV-negative women, followed by *Leptotrichia amnionii* in 38%, *Bacteroides ureolyticus* in 25%, and *Sneathia sanguinegens* in 19% of HPV positive cases (Table 2). The presence of the pathogens did not differ depending on the degree of the precancerous lesions or HPV type.

In the vaginal microbiome of five patients with confirmed ASCUS lesions and HPV positivity, we identified pathogens such as *Fusobacterium nucleatum*, *Gardnerella vaginalis*, *Atopobium vaginae*, *Pseudomonas aeruginosa*, *Ureaplasma parvum*, *Streptococcus mitis*, *Candida albicans*, *Prevotella bivia*, *Leptotrichia amnionii*, *Gardnerella vaginalis*, *Mycoplasma hominis*, *Sneathia sanguinegens*, *Prevotella disiens*, and *Parvimonas micra*. In the vaginal swab of six patients with LSIL lesions and a confirmed HPV positivity, we determined the presence of the following bacteria: *Gardnerella vaginalis*, *Atopobium vaginae*, *Mycoplasma hominis*, *Ureaplasma parvum*, *Bacteroides ureolyticus*, *Leptotrichia amnionii*, *Ureaplasma urealyticum*, *Sneathia sanguinegens*, *Parvimonas micra*, *Prevotella melaninogenica*, *Prevotella disiens*, *Streptococcus mitis*, *Aerococcus christensenii*, *Fusobacterium nucleatum*, *Prevotella intermedia*, *Prevotella nigrescens*, *Prevotella bivia*, *Corynebacterium aurimucosum*, *Streptococcus intermedius*, *Campylobacter gracilis*, and *Treponema denticola*. Furthermore, in one LSIL sample with HPV16 infection, no pathogenic bacteria were observed. However, in two specimens from LSIL, HPV-negative patients, we identified *Prevotella bivia*, *Leptotrichia amnionii*, *Ureaplasma parvum*, *Gardnerella vaginalis*, *Sneathia sanguinegens*, *Streptococcus mitis*, *Mobiluncus curtisii*, *Parvimonas micra*, and *Bacteroides ureolyticus*. Moreover, the following pathogens were confirmed in the vaginal swab of three HSIL, HPV-positive patients: *Ureaplasma parvum*, *Gardnerella vaginalis*, *Atopobium vaginae*, *Streptococcus mitis*, *Prevotella disiens*, *Prevotella bivia*, *Aerococcus christensenii*, *Leptotrichia amnionii*, *Bacteroides ureolyticus*, *Parvimonas micra*, and *Mobiluncus curtisii*. In one NILM, but HPV16-positive swab, we identified *Leptotrichia amnionii*, *Gardnerella vaginalis*, *Sneathia sanguinegens*, *Atopobium vaginae*, *Aerococcus christensenii*, *Prevotella disiens*, *Ureaplasma parvum*, and *Fusobacterium nucleatum*. In addition, in two NILM, HPV-negative patients, the vaginal microbiome diversity was low, and only *Gardnerella vaginalis* and *Ureaplasma parvum* were identified. On the other hand, in one vaginal swab from an NILM, HPV-negative woman, we identified *Prevotella bivia*, *Ureaplasma parvum*, *Prevotella disiens*, *Streptococcus agalactiae*, *Gardnerella vaginalis*, and *Mobiluncus mulieris*.

## 4. Discussion

Several works indicate the association of CST IV. and HPV infection, as well as the related development of cervical precancerous lesions. *L. iners*, which dominates CST III, is controversial. Its genome encodes proteins are important for maintaining the vaginal microbiome, such as FeS, a component of many redox systems. On the other hand, this microbe was also isolated from the vaginal flora of women with bacterial vaginosis, and its presence can lead to serious complications, such as premature birth, the development of endometriosis, and infection of the female reproductive tract [28]. One of the possible mechanisms underlying such complications is the higher ratio of the L form of lactic acid to its D form in women with CST III and CST IV, which leads to the activation of the inducer of extracellular matrix metalloproteinases. This increased activity alters cervical integrity and facilitates the entry of HPV into keratinocytes of the basement membrane [29]. Qingqing et al. [30] compared cervicovaginal microbiota composition between patients with persistent HPV infection, patients with transient infection, and healthy women. The authors demonstrated that the CST IV genus members *Prevotella*, *Sphingomonas*, and *Anaerococcus* were correlated with HPV persistent infection, while *L. iners* was correlated with transient infection [30]. In our work, we also confirmed the presence of genus *Prevotella* in the majority of hrHPV positive samples, albeit for both persistent and transforming infections. 

Nicolò et al. [31] demonstrated that *L. gasseri* and *L. jensenii* support eradication of cervicovaginal HPV infection, as these species provide a strong stimulus for IFN-γ production [31]. On the other hand, IL-17 was found to suppress immune system responses to HPV infection [32]. The same article demonstrated that IL-17 production is induced by the bacteria associated with vaginal dysbiosis [31]. 

Wei et al. [33] compared the vaginal microbiomes in HPV-positive and -negative cohorts, showing that the genus *Lactobacillus* dominates in both groups. However, the abudance of this genus was 10.3% lower in the group of HPV-positive patients (96.57% vs. 86.34%). The abudance of genus *Gardnerella* was almost nine-fold higher in the HPV positive group, compared to that among HPV negative women (6.42% vs. 0.72%), while settlement of the genus *Prevotella* was more than five times higher (2.44% vs. 0.44%). Other microbes associated with CST IV, such as *Megasphaera* and *Atopobium*, were also found in a higher quantity in the vaginal microbiomes of HPV-positive women, compared to the HPV negative group [33]. This result is in line with our work, where these genera were observed in HPV-positive women. Chao et al. [34] described the predominance of *Prevotella bivia*, *Enterococcus durans*, and *Porphyromonas uenonis* in the vaginal microbiome related to persistent HPV infection [34]. Usyk et al. [35] reported HPV infection development in two patients during follow-up visits. The authors recorded the presence of *Gardnerella* in the cervicovaginal swabs at the first visit as a biomarker for hrHPV progression, while the abundance of *L. iners* was associated with the clearance of the HPV infection [35]. 

Community state types IV and III are often associated with the presence of HPV infection in women, as well as the development of precancerous lesions. A study of 70 women with cervical intraepithelial lesions (CINs) and 50 healthy women showed a higher diversity of the vaginal microbiome in women with CINs, compared to that in controls. There was an increased risk of developing CIN observed in hrHPV-positive women with *Anaerococcus vaginae*, *Garderella vaginalis*, and *L. iners* identified in the vaginal microbiome, alongside a simultaneous decrease in the representation of *L. crispatus*, compared to that in hrHPV-negative group [36]. Our work also suggests the presence of *Garderella vaginalis* in hrHP- positive patients. Brotman et al. [37] examined the vaginal smears of 32 sexually active women for 16 weeks. HPV positivity was demonstrated in 71% of cases of women in CST III and 72% of cases of patients with CST IV. Additionally, a higher probability of the presence of HPV infection was demonstrated in women with CST III and CST IV. HPV clearance was described in this work as the transition from HPV negativity to positivity and back to the stage of HPV negativity. On the other hand, increased clearance of HPV was demonstrated in women with CST II *L. gasseri* and appears to be a potentially therapeutic species that can improve the clearance of HPV infection [37].

As previously mentioned, it is also possible to describe the relationship between the physiological lactobacillus settlement of the vagina and regression of HPV infection. *In vitro*, after treatment of HPV16-positive Caski cells with Lactobacillus supernatants, a reduction in the number of cells at the G2/M phase transition was observed. Additionally, a lower expression of mRNA E6 and E7 oncogenes was demonstrated and detected a more than six-fold reduction of E6 expression after 24 h of treatment with the dominant CST I microbe, *L. crispatus* [38]. Another study showed a decrease in the mRNA levels of HPV16 E6 and E7 oncoproteins after 24 and 48 h of incubation with *Bifidobacterium adolescentis* [39]. 

In our pilot study, the collection of samples is still ongoing; and we are increasing the number of participants to determine specific bacterial species and HPV genotype. Our study’s main limitations are the limited number of samples, differences in age, and phase of the menstrual cycle of the examined women, which can also affect the composition of the vaginal microbiome. On the other hand, the main strength of our work is that the microbial qPCR array that we used is able to identify 42 pathogenic bacterial species. In the next step, we are planning to investigate the colonization of the vagina with lactobacilli, which would allow us to evaluate bacterial vaginosis in the examined samples. The goal of our work is to point out the vaginal microbiome as a possible auxiliary tool in the diagnosis of cervical precancerosis. Some works also suggest that physiological correction of the composition of the vaginal microbiome may play an important role in the regression of the HPV infection of the cervix.

## 5. Conclusions

The HPV virus is responsible for almost all cases of cervical cancer. In our work, HPV16 dominated in patients with cervical precancerous lesions. We believe that there is an increased bacterial diversity of the vaginal microbiome in patients with cervical lesions, for which the HPV virus is largely responsible. In our study, the most common genera of bacteria were *Gardnerella*, *Atopobium*, *Prevotella*, *Ureaplasma*, *Leptotrichia*, *Bacteroides*, and *Sneathia*. Cervical cancer is easily avoidable with the available methods for both primary and secondary prevention; nevertheless, the prevalence of this cancer among the population is still high. We suggest that support for the physiological composition of vaginal microflora could become a widely available tool for preventing the development of cervical precancerous lesions.

## Figures and Tables

**Table 1:** The identified HPV genotypes in cervical swabs taken from women with different stage of cervical abnormality.

	*n*	HPV16 *n*	HPV33 *n*	HPV52 *n*	HPV66 *n*	HPV70 *n*	HPV18 + HPV66 *n*	Negat *n*
NILM	4	1						3
ASCUS	5	3	1		1			
LSIL	9	4			1	1	1	2
HSIL	3	2		1				
Total	21	10	1	1	2	1	1	5

NILM, negative for intraepithelial lesion or malignancy; ASCUS, atypical squamous cells of undetermined significance; LSIL, low-grade squamous intraepithelial lesion; HSIL, high-grade intraepithelial lesion; HPV, human papillomavirus.

**Table 2:** Vaginal microbiome composition in HPV positive and HPV negative cervical smears. (The presence of bacteria marked with “+”).

	*Gardnerella vaginalis*	*Atopobium vaginae*	*Prevotella* *Bivia*	*Ureaplasma parvum*/ *urealyticum*	*Leptotrichia amnionii*	*Bacteroides ureolyticus*	*Sneathia sanguinegens*
ASCUS, HPV16	+	+					
ASCUS, HPV66	+						
ASCUS, HPV33	+						
ASCUS, HPV16				+			
ASCUS, HPV16	+	+	+	+	+		+
LSIL, HPV18,66	+	+	+	+			
LSIL, HPV negat.	+		+	+	+		+
LSIL, HPV negat.	+					+	+
LSIL, HPV16						+	
LSIL, HPV16	+			+	+		
LSIL, HPV66	+		+	+	+	+	
LSIL, HPV16				+			
LSIL, HPV70	+	+	+	+	+	+	+
LSIL, HPV16							
HSIL, HPV16	+	+	+	+	+	+	
HSIL, HPV52	+	+	+				
HSIL, HPV16	+	+	+				
NILM, HPV negat.	+						
NILM, HPV negat.				+			
NILM, HPV negat.	+		+	+			
NILM, HPV16	+	+		+	+		+

ASCUS, atypical squamous cells of undetermined significance; LSIL, low-grade squamous intraepithelial lesion; HSIL, high-grade intraepithelial lesion; NILM, negative for intraepithelial lesion or malignancy; HPV, human papillomavirus; HPV negat., negative for the presence of HPV.

## Data Availability

The data presented in this study are available on request from the corresponding author.
